# 无冻存一体化自体造血干细胞移植模式治疗新诊断多发性骨髓瘤的回顾性临床研究

**DOI:** 10.3760/cma.j.cn121090-20230929-00152

**Published:** 2024-05

**Authors:** 曦 杨, 成龙 李, 姣 陈, 菲菲 车, 蓉 肖, 慧 李, 娟 黄, 涛 姜, 海清 杨, 欢 王, 小串 况, 晓兵 黄

**Affiliations:** 电子科技大学附属医院（四川省人民医院）血液科，成都 610071 Department of Hematology, Sichuan Provincial Peoples Hospital, Affiliated Hospital of University of Electronic Science and Technology of China, Chengdu 610071, China

**Keywords:** 多发性骨髓瘤, 自体造血干细胞移植, 无冻存一体化移植模式, Multiple myeloma, Autologous hematopoietic stem cell transplantation, Non-cryopreserved integrated transplantation model

## Abstract

**目的:**

探讨无冻存一体化自体造血干细胞移植模式在多发性骨髓瘤（MM）患者中的疗效和安全性。

**方法:**

纳入2020年7月31日至2022年12月31日在电子科技大学附属医院四川省人民医院接受自体造血干细胞移植的新诊断多发性骨髓瘤（NDMM）患者96例，对其临床资料进行回顾性分析。41例患者接受无冻存一体化移植模式（观察组），造血干细胞动员采集后冷藏于医用输血冰箱（4 °C）并立即启动美法仑预处理，预处理结束24 h后回输自体造血干细胞；55例患者接受传统移植模式（对照组），造血干细胞动员采集后液氮冷冻保存，择期启动移植流程。两组患者均采用G-CSF联合普乐沙福进行自体造血干细胞动员。

**结果:**

①观察组移植前疾病状态为非常好的部分缓解（VGPR）及完全缓解（CR）患者占比显著高于对照组［82.9％（34/41）对60.0％（33/55），*P*＝0.016］。②与对照组相比，观察组1级口腔黏膜炎的发生率较高（*P*<0.001），但2、3级口腔黏膜炎的发生率较低（*P*＝0.004，*P*＝0.048），两组均未发生≥4级口腔黏膜炎；观察组1级腹泻的发生率较高（*P*＝0.002），3级腹泻的发生率较低（*P*＝0.007），4级腹泻的发生率差异无统计学意义（*P*＝0.506），两组均未发生5级腹泻。③观察组细菌感染发生率低于对照组（34.1％对65.5％，*P*＝0.002），真菌感染（29.3％对31.4％，*P*＝0.863）、病毒感染（4.88％对3.64％，*P*＝0.831）发生率差异无统计学意义。④观察组与对照组粒细胞植入时间和血小板植入时间差异无统计学意义［10（8～20）d对11（8～17）d，*P*＝0.501；13（10～21）d对15（10～20）d，*P*＝0.245］。⑤移植后100 d前所有患者均未使用来那度胺治疗。移植后30 d，观察组CTL、NK、Th细胞计数低于对照组（*P*<0.001，*P*＝0.049，*P*＝0.002），NKT细胞计数高于对照组（*P*＝0.024）。移植后100 d，观察组CTL、NKT、Th细胞计数高于对照组（*P*＝0.025，*P*＝0.011，*P*＝0.007），NK细胞计数两组差异无统计学意义（*P*＝0.396）。⑥中位随访18（4～33）个月，观察组和对照组移植后2年总生存率分别为91.5％、78.2％（*P*＝0.337），无复发生存率分别为85.3％、77.6％（*P*＝0.386），累积复发率分别为9.8％、16.9％（*P*＝0.373）。

**结论:**

无冻存一体化自体造血干细胞移植模式在NDMM中可获得与传统移植模式相似的疗效，重度黏膜炎和感染的发生率低于传统移植模式。

## Abstract (en)

**Objective:**

To explore the efficacy and safety of cryopreservation-free integrated autologous hematopoietic stem cell transplantation（HSCT）model for patients with multiple myeloma.

**Methods:**

A total of 96 patients with newly diagnosed multiple myeloma（NDMM）between July 31, 2020, and December 31, 2022, were retrospectively analyzed, of which 41 patients in the observation group received integrated non-cryopreserved transplantation mode. After hematopoietic stem cells were mobilized and collected, melphalan was started immediately for pre-transplant conditioning, and non-cryopreserved grafts from the medical blood transfusion refrigerator were directly injected intravenously into the patient within 24–48 h after the melphalan conditioning. The control group consisted of 55 patients who received traditional transplantation mode. After hematopoietic stem cells were collected, stem cell cryopreservation was performed in liquid nitrogen, and then the transplant plans were started at the right time. All patients received mobilization of autologous hematopoietic stem cells using the G-CSF combined with the plerixafor.

**Results:**

① A total of 34 patients（82.9％）with VGPR plus CR in the observation group were significantly higher than 33 patients（60.0％）in the control group（*P*＝0.016）. ② Compared with the control group, the incidence of grade 1 oral mucosal inflammation was higher in the observation group（*P*<0.001）; however, the incidence of grades 2 and 3 oral mucosal inflammation was lower（*P*＝0.004, *P*＝0.048）, and neither group experienced grade 4 or above oral mucosal inflammation. The incidence of grade 1 diarrhea was higher in the observation group（*P*＝0.002）, whereas the incidence of grade 3 diarrhea was lower（*P*＝0.007）. No statistically significant difference was observed in the incidence of grade 4 diarrhea（*P*＝0.506）, and neither group experienced grade 5 diarrhea. ③ The incidence of bacterial infection in the observation group was lower than that in the control group（34.1％ *vs* 65.5％, *P*＝0.002）, whereas no statistically significant difference was observed in the incidence of fungal infection（29.3％ *vs* 31.4％, *P*＝0.863）and viral infection（4.88％ *vs* 3.64％, *P*＝0.831）. ④ No statistically significant difference was observed in the implantation time of granulocytes and platelets between the observation and control groups［10（8–20）days *vs* 11（8–17）days, *P*＝0.501; 13（10–21）days *vs* 15（10–20）days, *P*＝0.245］. ⑤ All patients did not receive lenalidomide treatment 100 days post-transplantation. At 30 days post-transplantation, the CTL, NK, and Th cell counts in the observation group were lower than those in the control group（*P*<0.001, *P*＝0.002, *P*＝0.049）, and the NKT cell counts were higher than those in the control group（*P*＝0.024）. At 100 days post-transplantation, the CTL, NKT, and Th cell counts in the observation group were higher than those in the control group（*P*＝0.025, *P*＝0.011, *P*＝0.007）, and no statistically significant difference in NK cell counts was observed between the two groups（*P*＝0.396）. ⑥ The median follow-up was 18（4–33）months. The overall 2-year survival rates of the observation and control groups post-transplantation were 91.5％ and 78.2％, respectively（*P*＝0.337）. The recurrence-free survival rates were 85.3％ and 77.6％, respectively（*P*＝0.386）, and the cumulative recurrence rates were 9.8％ and 16.9％, respectively（*P*＝0.373）.

**Conclusion:**

In NDMM, the cryopreservation-free integrated autologous HSCT model can achieve similar therapeutic effects as traditional transplantation models, with lower rates of severe mucosal inflammation and infection compared with traditional transplantation models.

无论在传统化疗时代，还是在当前的新药时代，自体造血干细胞移植（auto-HSCT）均为新诊断多发性骨髓瘤（NDMM）患者的一线治疗选择[1]–[3]。目前，接受auto-HSCT治疗的中国NDMM患者比例显著低于发达国家[4]–[6]。当前，在我国NDMM患者中开展auto-HSCT受限原因大致如下：①对于auto-HSCT在MM治疗中的重要性认识不足；②造血干细胞采集不达标或失败，疾病缓解不佳，治疗时间过长。③造血干细胞冷冻保存条件的限制，很多基层医院难以获得超低温冰箱（至少零下80 °C）或液氮保存造血干细胞的条件。④传统造血干细胞移植流程中，造血干细胞采集、冻存、转运、复苏到回输等繁琐过程不仅增加了移植成本，也一定程度上抑制了地方开展auto-HSCT的积极性。我中心利用造血干/祖细胞体外生存时间长于移植预处理化疗时间的特点，探索开展“无冻存一体化自体造血干细胞移植”模式（造血干细胞动员采集后立即启动预处理化疗，化疗结束24 h后直接回输冷藏于医用输血冰箱的自体造血干细胞）。本研究对无冻存一体化模式与传统模式auto-HSCT治疗NDMM的疗效及安全性进行回顾性对比分析。

## 病例与方法

一、纳入患者基本信息

本研究纳入2020年7月31日至2022年12月31日期间在电子科技大学附属医院四川省人民医院血液科接受auto-HSCT治疗的96例NDMM患者，其中41例接受无冻存一体化移植模式（观察组），造血干细胞动员采集后冷藏于医用输血冰箱并立即启动美法仑预处理，预处理结束24 h后直接回输冷藏的自体造血干细胞；55例接受传统移植模式（对照组），造血干细胞动员采集后液氮冷冻保存，择期启动移植。两组患者的临床信息详见表1。

二、自体造血干细胞动员及采集

观察组和对照组动员均采用粒细胞集落刺激因子（G-CSF）联合普乐沙福的稳态动员方案：聚乙二醇化粒细胞集落刺激因子6 mg皮下注射（第1天）、G-CSF 5 µg/kg每日2次皮下注射（第1～5天）、普乐沙福20 mg皮下注射（第4天晚22时），第5天早8时使用德国费森尤斯血细胞分离机COM-TEC的单个核细胞采集程序进行采集自体造血干细胞。采集目标：对照组CD34^+^细胞>2.0×10^6^/kg；观察组CD34^+^细胞>1.0×10^6^/kg。两组均要求总有核细胞计数（TNC）>（6.0～8.0）×10^8^/kg。造血干细胞采集结束后，观察组的采集物立即置于医用冰箱4 °C冷藏保存；对照组的采集物液氮冷冻保存。

三、auto-HSCT及移植后治疗流程

96例患者中82例采用美法仑200 mg/m^2^预处理化疗，个别因年龄、心功能、肾功能等原因减量至140 mg/m^2^，其中观察组6例（14.6％），对照组8例（14.6％）。移植过程中预防感染策略如下：头孢噻肟预防细菌感染，阿昔洛韦预防病毒感染，泊沙康唑肠溶片预防真菌感染。观察组患者在外周造血干细胞采集结束后立即药浴入仓，并马上启动美法仑预处理方案，化疗结束24 h后将采集物从医院输血中心医用冰箱（4 °C冷藏）取回后直接回输。对照组患者择期药浴入仓，启动美法仑预处理化疗，化疗结束24 h后将采集物从四川省脐带血干细胞库取回，复苏后回输。无论是否巩固治疗，自移植后100 d开始，均予以来那度胺维持治疗（25 mg/d，第1～21天），28 d为1个疗程，不能耐受的患者减量为10 mg/d。

四、免疫重建指标的检测

本研究关注观察组与对照组在免疫重建方面的差异，进行了随访监测。监测时间点为拟行移植前、移植后30 d、移植后100 d。监测的免疫指标包括辅助性T细胞（Th，CD3^+^ CD4^+^ CD8^−^）、细胞毒性T淋巴细胞（CTL，CD3^+^CD4^−^CD8^+^）、自然杀伤性T细胞（NKT，CD3^+^CD16^+^CD56^+^）、自然杀伤细胞（NK，CD3^−^CD16^+^CD56^+^）。

五、不同感染类型的诊断标准

细菌感染标准包括：①存在感染临床症状（如发热、咳嗽、尿频尿痛、腹痛腹泻等）；②炎症相关指标显著升高（C反应蛋白>100 mg/L或降钙素原>0.5 mg/L）；③病原学检测结果提示细菌感染；④抗细菌治疗后感染症状控制、炎症指标下降。满足④和另外三项中的任何一项可定义为细菌感染。真菌感染参照《血液病/恶性肿瘤患者侵袭性真菌病（IFD）的诊断标准与治疗原则（第六次修订版）》[7]中的临床诊断或拟诊标准。所有患者经抗真菌治疗后感染症状控制、影像学改善、炎症指标下降。EB病毒DNA（EBV-DNA）>5000拷贝/ml定义为EBV感染，巨细胞病毒DNA（CMV-DNA）>5000拷贝/ml定义为CMV感染。

六、不良反应及MM疗效评价

参照NCI-CTCAE version 4.03分级标准，口腔黏膜炎和腹泻均分为5级。所有患者auto-HSCT后规律随访，定期评估疗效。评估时间点为移植后30 d、100 d、180 d、365 d，直至疾病复发。疗效评价标准参照中国多发性骨髓瘤诊治指南（2022修订版）[8]。

七、随访

采用电话、查阅住院/门诊病历方式获得随访资料，随访截止日期为2023年4月30日，中位随访18（4～33）个月。

八、统计学处理

采用独立样本*t*检验或卡方检验进行两组间基线特征、不良反应发生率及免疫重建指标比较。采用非参数秩和检验比较两组间植入时间的差异。采用生存分析法评估移植后总生存（OS）、无复发生存（RFS）、累积复发率（CIR）、移植相关死亡率（TRM）等生存指标。*P*值小于0.05认为差异具有统计学意义。

## 结果

一、临床特征

所有患者移植前均接受诱导化疗，包括VRD方案（硼替佐米+来那度胺+地塞米松）74例、VCD方案（硼替佐米+环磷酰胺+地塞米松）10例、DVD方案（脂质体多柔比星+长春新碱+地塞米松）11例、DKD方案（达雷妥尤单抗+卡非佐米+地塞米松）1例。观察组移植前34例（82.9％）患者获得VGPR以上疗效（VGPR+CR），显著高于对照组的33例（60.0％）（*P*＝0.016），其他临床指标两组之间差异无统计学意义（表1）。

**表1:** 接受无冻存一体化（观察组）和传统模式（对照组）自体造血干细胞移植的新诊断多发性骨髓瘤患者临床特征比较

基线特征	观察组（41例）	对照组（55例）	统计量	*P*值
移植年龄［岁，*M*（范围）］	55（48~69）	54（44~66）	1.301	0.202
性别［例（%）］				
女	21（51.2）	27（49.1）	0.042	0.843
男	20（48.8）	28（50.9）	0.043	0.843
疾病亚型［例（%）］				
IgG	19（46.3）	24（43.6）	0.072	0.791
IgA	10（24.4）	12（21.8）	0.091	0.772
IgM	1（2.44）	2（3.64）	0.071	0.802
IgD	4（9.76）	3（5.45）	0.161	0.692
轻链型	5（12.2）	12（21.8）	1.492	0.223
双克隆	2（4.88）	2（3.64）	0.051	0.832
细胞遗传学及分子遗传学改变［例（%）］				
t(11;14)或t(6;14)	5（12.2）	9（16.4）	0.332	0.572
t(14;16)	3（7.32）	5（9.09）	0.004	0.953
t(14;20)	3（7.32）	2（3.64）	0.112	0.742
t(4;14)	7（17.1）	8（14.5）	0.113	0.742
1q21扩增	8（19.5）	9（16.4）	0.162	0.692
del(17p)或TP53突变	5（12.2）	8（14.5）	0.113	0.742
危险分层［例（%）］				
Durie-Salmon Ⅲ期	16（39.0）	22（40.0）	0.009	0.923
R-ISS Ⅲ期	14（34.1）	19（34.5）	0.002	0.961
Mayo mSMART3.0分层高危	25（60.0）	31（56.4）	0.211	0.652
移植前化疗疗程［个，*M*（范围）］	4（3~9）	6（4~13）	0.022	0.423
移植前疾病状态［例（%）］				
CR	19（46.3）	15（27.3）	3.732	0.053
VGPR	15（36.6）	18（32.7）	0.163	0.694
PR	6（14.6）	17（30.9）	3.423	0.065
PD	1（2.44）	5（9.09）	0.823	0.372
TNC回输量［×10^8^/kg，*M*（范围）］）	17.9（6.4~57.6）	19.6（5.2~60.3）	−0.742	0.464
CD34^+^细胞回输量［×10^6^/kg，*M*（范围）］	3.3（1.2~7.9）	3.7（1.6~13.3）	−1.262	0.213

**注** R-ISS：修订的国际分期系统；CR：完全缓解：PR：部分缓解；VGPR：非常好的部分缓解；PD：疾病进展；TNC：总有核细胞

二、移植相关并发症

与对照组相比，观察组1级口腔黏膜炎的发生率较高（*P*<0.001），但2、3级口腔黏膜炎的发生率较低（*P*＝0.004，*P*＝0.048），两组均未发生≥4级口腔黏膜炎，详见表2。观察组1级腹泻的发生率较高（*P*＝0.002），3级腹泻的发生率较低（*P*＝0.007），4级腹泻的发生率差异无统计学意义（*P*＝0.506），两组均未发生5级腹泻，详见表3。观察组细菌感染发生率低于对照组（34.1％对65.4％，*P*＝0.002），真菌感染、病毒感染发生率差异无统计学意义（29.2％对30.9％，*P*＝0.863；4.9％对3.6％，*P*＝0.831），详见表4。

**表2:** 无冻存一体化（观察组）和传统模式（对照组）自体造血干细胞移植新诊断多发性骨髓瘤患者口腔黏膜炎的发生情况比较［例（％）］

组别	例数	1级	2级	3级
观察组	41	26（63.4）	14（34.1）	1（2.4）
对照组	55	12（21.8）	35（63.6）	8（14.5）

*χ*^2^值		16.990	8.170	4.050
*P*值		<0.001	0.004	0.048

**表3:** 无冻存一体化（观察组）和传统模式（对照组）自体造血干细胞移植新诊断多发性骨髓瘤患者腹泻发生情况比较［例（％）］

组别	例数	1级	2级	3级	4级
观察组	41	25（60.9）	14（34.1）	2（4.9）	0（0）
对照组	55	16（29.1）	23（41.8）	14（25.4）	2（3.6）

*χ*^2^值		9.760	0.580	7.160	/
*P*值		0.002	0.445	0.007	0.506

**注** /：Fisher精确概率法

**表4:** 无冻存一体化（观察组）和传统模式（对照组）自体造血干细胞移植新诊断多发性骨髓瘤患者感染发生情况比较［例（％）］

组别	例数	细菌感染	真菌感染	病毒感染
观察组	41	14（34.1）	12（29.2）	2（4.9）
对照组	55	36（65.4）	17（30.9）	2（3.6）

*χ*^2^值		9.230	0.030	0.460
*P*值		0.002	0.863	0.831

三、造血重建

对照组、观察组中位TNC回输量分别为19.6（5.2～60.3）×10^8^/kg、17.9（6.4～57.6）×10^8^/kg（*P*＝0.464），CD34^+^细胞回输量分别为3.3（1.2～7.9）×10^6^/kg、3.7（1.6～13.3）×10^6^/kg（*P*＝0.213）。所有96例患者均顺利完成造血重建。观察组、对照组中位粒细胞植入的中位时间为10（8～20）d、11（8～17）d（*z*＝0.674，*P*＝0.501），血小板植入的中位时间13（10～21）d、15（10～20）d（*z*＝1.164，*P*＝0.245）。

四、免疫重建

移植后100 d前所有患者均未使用来那度胺治疗。移植后30 d，观察组CTL、NK、Th细胞计数低于对照组（*P*<0.001，*P*＝0.002，*P*＝0.049），NKT细胞计数高于对照组（*P*＝0.024），详见表5。移植后100 d，观察组CTL、NKT、Th细胞计数高于对照组（*P*＝0.025，*P*＝0.011，*P*＝0.007），NK细胞计数两组差异无统计学意义（*P*＝0.396），详见表6。

**表5:** 无冻存一体化（观察组）和传统模式（对照组）自体造血干细胞移植新诊断多发性骨髓瘤患者移植后30 d免疫重建指标比较（*x*±*s*）

组别	例数	CTL细胞（×10^8^/L）	NK细胞（×10^8^/L）	NKT细胞（×10^7^/L）	Th细胞（×10^8^/L）
观察组	41	6.64±4.69	1.69±1.79	3.76±3.75	1.88±1.29
对照组	55	10.48±4.79	2.61±1.44	2.39±2.71	2.29±0.49

*t*值		−5.243	−3.291	2.339	−2.035
*P*值		<0.001	0.002	0.024	0.049

**注** CTL细胞：细胞毒性T淋巴细胞；NK细胞：自然杀伤细胞；NKT细胞：自然杀伤性T细胞；Th细胞：辅助性T细胞

**表6:** 无冻存一体化（观察组）和传统模式（对照组）自体造血干细胞移植新诊断多发性骨髓瘤患者移植后100 d免疫重建指标比较（*x*±*s*）

组别	例数	CTL细胞（×10^8/^L）	NK细胞（×10^8^/L）	NKT细胞（×10^7^/L）	Th细胞（×10^8^/L）
观察组	41	14.36±12.92	1.69±1.79	6.22±6.19	3.59±2.29
对照组	55	9.66±4.29	1.45±1.57	3.63±3.54	2.58±1.63

*t*值		2.329	0.859	2.679	2.824
*P*值		0.025	0.396	0.011	0.007

**注** CTL细胞：细胞毒性T淋巴细胞；NK细胞：自然杀伤细胞；NKT细胞：自然杀伤性T细胞；Th细胞：辅助性T细胞

四、生存与复发

所有患者中位随访18（4～33）个月，观察组38例均存活（92.7％），对照组50例存活（90.9％）；对照组有10例（18.2％）复发，观察组仅4例（9.8％）复发；对照组9例死亡，其中7例死于复发；观察组3例死亡，均死于复发。观察组和对照组移植后2年OS率分别为91.5％、78.2％（*P*＝0.337）（图1A），RFS率分别为85.3％、77.6％（*P*＝0.386）（图1B），CIR分别为9.8％、16.9％（*P*＝0.373）（图1C）。

**图1:**
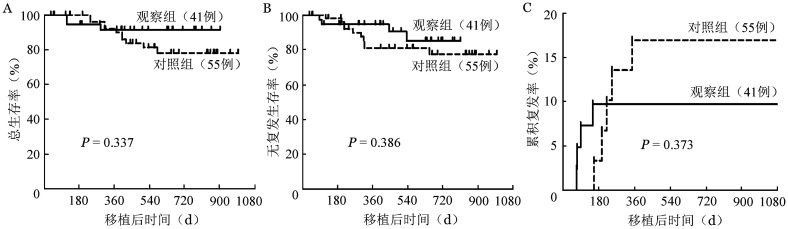
无冻存一体化（观察组）和传统模式（对照组）组自体造血干细胞移植后总生存曲线（A）、无复发生存曲线（B）和复发曲线（C）

## 讨论

无冻存一体化移植模式可使更多NDMM患者早期接受移植，从而在一线巩固时达到深层缓解，获得更长的无复发生存时间（RFS）。IFM2009研究显示，对于NDMM作为一线巩固的早期移植相较于复发后进行的延后移植，达到MRD阴性比例以及PFS显著升高，预示患者生活质量可以得到明显改善[9]。所以早期移植尽管无法显著延长OS时间，但仍建议作为一线巩固的重要组成环节。

MM患者auto-HSCT的主要难点之一是如何动员采集足量的造血干/祖细胞以保证顺利植入。本研究发现，在无冻存一体化移植模式下，CD34^+^细胞计数只要大于1×10^6^/kg，即使低于2×10^6^/kg，依然可以保证和传统模式相似的造血植入程度。尽管目前病例数相对减少，但这一趋势非常明显。其原因可能是由于移植物在短时间冷藏的情况下可保留足够单个核细胞活性，从而保证了造血干细胞在较低数量的情况下顺利植活，而未必需要满足指南推荐2.0×10^6^/kg。由此可见，无冻存一体化移植模式似乎可以降低造血植入所需CD34^+^细胞数量要求，减轻动员难度，有待于更多病例进一步证实。

重度黏膜炎和感染是MM患者auto-HSCT面临的两大主要并发症，尤其是大剂量美法仑导致肠道黏膜屏障破坏，肠道微生物移行引起的机会性感染。相较于传统移植模式的对照组，采用无冻存一体化移植模式的观察组重度黏膜炎、细菌感染发生率显著降低。可能的机制是：①移植物中大量免疫活性细胞保留促进了黏膜屏障的修复，同时具有一定抗感染作用；②感染率降低，导致抗生素使用减少，有利于术后肠道微生态恢复，从而促进了黏膜屏障修复。这些机制有待于进一步阐明。

auto-HSCT后免疫重建对于MM患者临床预后具有重要影响。目前国内外指南和权威文献认为造血干细胞冻存对auto-HSCT后免疫重建、生存获益的影响并不明确[9]–[11]。我们前期的观察以及有关研究发现，移植物冻存会导致CD3^+^细胞数量和功能的严重损伤[12]–[14]。一项来自于梅奥诊所的研究[15]进一步证实了MM患者auto-HSCT后免疫重建与患者预后有明显的相关性。该研究纳入58例MM患者，移植前均提前进行自体造血干细胞采集并冻存备用；通过定期监测移植后60 d～100 d细胞因子、免疫细胞组分变化情况，结合临床指标，发现富集衰老和耗竭T细胞表型的患者群体具有更高的复发率、更快的疾病进展率以及更短的生存期。另外一篇文献分析了移植后3个月内NK细胞的恢复情况与MM预后的关系[16]。该研究发现，MM患者auto-HSCT后90 d的MRD阴性患者中NK细胞绝对计数（131.38 /µl）显著高于MRD阳性组（43.86 /µl），提示NK细胞的免疫重建有助于移植后免疫介导的肿瘤细胞清除。上述两项研究均采用传统的移植模式。本研究中与传统移植模式相比，观察组的移植物未经冻存，在体外短时间冷藏下仍含有丰富的Th、CTL、NK、NKT细胞等活性免疫细胞，可能有利于快速免疫重建。

本研究中，两组病例在造血重建、生存获益方面差异无统计学意义，但移植后早期免疫重建模式存在明显不同——突出表现在观察组移植后100 d时NKT细胞、CD4^+^细胞及CD8^+^细胞显著增多，这将为移植后巩固和维持治疗提供更好基础，能否带来长期生存获益尚需继续观察。无冻存一体化移植模式还避免了回输冻存干细胞产品中细胞保护液二甲基亚砜所带来的不良反应、省去移植物冻存和复苏费用，重度黏膜炎和感染率也低于传统移植模式。因此，无冻存一体化移植模式尤其有利于auto-HSCT在经济欠发达地区的推广。

本研究仍存在一些局限性：①如患者首次采集干细胞失败或动员过程中出现感染，则无法按该模式序贯移植；②该模式需要医务人员对动员、采集、干细胞保存、移植等流程进行整体统筹安排，其中任何一个环节出现问题，则无法顺利完成；③该模式主要适用于NDMM的一线巩固。当前MM新药层出不穷，那些移植前已获得高灵敏度MRD阴性的患者是否适用该移植模式，还需要开展前瞻性的临床研究进一步证实。
